# Recombinant and Plasma-Purified Human C1 Inhibitor for the Treatment of Hereditary Angioedema

**DOI:** 10.1097/1939-4551-3-S3-S29

**Published:** 2010-09-15

**Authors:** Michael M Frank

**Affiliations:** 1 Duke University Medical Center, Durham, North Carolina

**Keywords:** HAE, contact system, C1INH

## Abstract

**Background**

Agents for prophylaxis of hereditary angioedema (HAE) have been available in the United States for several decades, but their usefulness is limited by side effects and they cannot be used at all in some patients. No agents have been available in the United States to specifically treat acute attacks. HAE types I and II are associated with low functional levels of C1 inhibitor, and evidence accumulated over decades suggests that intravenous infusion of C1 inhibitor is useful for terminating angioedema attacks and for prophylaxis.

**Key Points**

C1 inhibitor derived from pooled human plasma has been available for decades in Europe, and 2 preparations have been recently introduced into the United States. Both have been efficacious in carefully controlled double-blind studies. One preparation, Cinryze, was approved by the U.S. Food & Drug Administration (FDA) for prophylaxis of HAE attacks in October 2008, and the second, Berinert, was approved by the FDA for treatment of acute attacks in October 2009. A third preparation, the recombinant human C1 inhibitor Rhucin, is completing clinical trials. Although not yet approved by the FDA, preliminary results made available suggest that Rhucin, too, is effective in terminating attacks of HAE.

**Conclusions**

Americans will now have access to effective acute and prophylactic treatments with C1 inhibitor for hereditary angioedema.

## 

Hereditary angioedema (HAE) is an episodic swelling disorder that involves the subcutaneous tissues of the extremities or the mucosa of the bowel and occasionally the tissues of the face, mouth, and pharynx or the genital area[1,2] Attacks most commonly increase in severity for about 1.5 days and then resolve during about the same period of time. Patients are usually most bothered by the painful abdominal attacks, which can be very severe and can lead to abdominal surgery; however, the development of laryngeal angioedema can lead to asphyxiation and death. HAE types I and II are caused by mutations in 1 of the 2 gene alleles that code for the plasma protein C1 inhibitor. This protein was named for its ability to regulate the activity of the complement protein C1, but C1 inhibitor has been found to act as a regulator or inhibitor of the clotting, fibrinolytic, and kinin-generating systems in plasma as well. It is now believed that complement is not the major system responsible for HAE attacks, although the failure of C1 inhibitor to regulate activation of the complement system in these patients has led to the most commonly used diagnostic test other than the level or function of C1 inhibitor itself, the plasma concentration of C4; the level of the complement protein C4 is almost always low in these patients. The failure to adequately regulate the kinin system leads to the unregulated formation of bradykinin, which in turn leads to angioedema[3].

Identification of the elements of the kinin pathway has led to the development of highly specific therapy; however, even before this information was available, considerable progress was made in attempts to provide long-term prophylaxis for patients with HAE. Epsilon aminocaproic acid, the first agent found to be effective in prophylactic treatment of HAE,[4,5] was identified during a random screen of diseases that may respond to treatment with this agent. Frank and colleagues at the National Institutes of Health performed a small double-blind trial that confirmed the effectiveness of the drug in preventing HAE[6]. Over time, it became clear that epsilon aminocaproic acid, a fibrinolysis inhibitor, has many side effects that limit its use. A related drug, tranexamic acid, was developed that is less toxic and has been used with success in Europe, but it has not been widely used in the United States[7]. Several years after the identification of the antifibrinolytics as useful in prophylaxis, the androgens were shown to be effective in the prophylaxis of HAE[8]. Their mechanism of action is still not completely clear, but patients with HAE are heterozygous for the defective C1 inhibitor gene allele, and androgens seem to increase synthesis of C1 inhibitor by the normal gene allele. Androgens have proven very useful, but they are not effective in everyone, cannot be used in children or pregnant women, and have a great many side effects that, although generally mild, preclude their use in some people[9]. Furthermore, they regularly induce weight gain and alterations in blood lipids that may predispose patients taking them for decades to atherosclerosis and cardiovascular disease[10]. None of these prophylactic drugs are effective as acute therapy and it is clear that new, more-specific agents are needed. Fresh-frozen plasma has been widely used in acute attacks[11]. However, it rarely can increase angioedema during attacks because it supplies the precursor proteins for kinin generation.

## Development of C1 Inhibitor Therapy

In 1963, Donaldson and Evans reported that HAE patients have a specific inherited defect in the plasma protein C1 inhibitor[12]. Patients with type I HAE were later shown to have low levels of this protein because its synthesis or secretion was prevented by mutations in one of the gene alleles; in general, patients with type II HAE have a defect in a gene allele that results in secretion of a nonfunctioning protein[13]. Infusion of the missing functional protein C1 inhibitor was an obvious approach to specific therapy. Three groups, the American Red Cross, the Dutch Red Cross, and the Behring Company in Germany all set out to purify C1 inhibitor from plasma. All of these groups already had facilities for collecting and pooling plasma from thousands of donors and purifying individual plasma proteins because all were suppliers of gamma globulin for patient use. The author's own group had access to the American Red Cross preparation and in 1980 reported that C1 inhibitor infusion raised plasma C1 inhibitor levels as expected in 8 patients and, as a consequence of its action in inhibiting activated C1, also raised the levels of serum C4[14]. We reported that this treatment terminated angioedema attacks in 5 of our patients who were having attacks at the time of their infusion[14]. At about the same time, the Dutch Red Cross developed a preparation of C1 inhibitor, and Agostoni et al reported that it was effective in terminating attacks of angioedema[15]. Bork and Witzke also reported that the German preparation was effective in terminating an attack of HAE[16].

## Development of the Currently Used C1 Inhibitor Preparations

In 1980, the importance of blood transfusion in transmitting the newly discovered disease AIDS became clear. The American Red Cross turned its attention to developing procedures to make the blood supply safe, and they prepared no further batches of C1 inhibitor. However, both the Dutch Red Cross and Behring continued to prepare batches of C1 inhibitor for therapeutic purposes. The manufacturing arm of the Dutch Red Cross was merged into the company Sanquin in 2003. In 1989, heat treatment was added to the preparation of this product, and now nanofiltration has also been added as an extra step to ensure the absence of contaminating viruses. The Behring product was first licensed as a unpasteurized product in Germany in 1979 and as a pasteurized product in 1985. Thus, these 2 products are available in Europe and have been used in patients for decades and, since the introduction of further purification steps 2 decades ago, have not been noted to transmit any infectious agents. The purification procedure in both cases is designed to remove viral contaminants. Some years after the initial reports of successful therapy, the Austrian company Immuno, another supplier of gamma globulin, also began to prepare a C1 inhibitor and to make it available in Europe. Waytes et al examined this preparation in a double-blind study reported in 1996 and demonstrated that it was effective for both prophylaxis and acute treatment of angioedema attacks[17]; however, this product is no longer available. Many subsequent reports have testified to the efficacy of the European preparations. In particular, Bork and colleagues have published extensively on the effect of the Behring preparation in terminating acute attack[18,19]. Because prevention using attenuated androgens was less common in Germany, an enormous number of attacks were treated. For example, Bork et al reported the effect of C1 inhibitor on symptoms in thousands of HAE abdominal attacks. Many patients began to recover at about 30 minutes, and by 1 hour after treatment began most patients were well on the road to recovery[19].

## Introduction of C1 Inhibitor Into the American Market

Several years ago, Behring and Sanquin made the decision to bring their European plasma-derived products to the United States. Over the years the original Behring Company was first divided and then acquired by a number of companies, and now it is part of the larger company CSL Behring. The pharmaceutical company Lev was organized in the United States to test and bring to market the Sanquin product. More recently, Lev was acquired by Viropharma.

## Recombinant C1 Inhibitor

A new approach for the development of a human C1 inhibitor for treatment of HAE attacks was developed by a second Dutch company, Pharming[20]. This company pioneered the concept of isolating the human gene for a plasma protein and fusing it to a bovine casein promoter. The new gene was transduced into rabbits, and the animals secrete the human gene product in their milk after pregnancy and delivery. The human protein is then isolated from the milk. This company is in the late stages of testing their product, called Rhucin, in treatment of HAE attacks[21].

## Testing of the New Preparations

A series of double-blind studies of treatment of acute attacks were initiated in the United States to fulfill Food and Drug Administration (FDA) requirements. The studies performed had much in common. All were placebo-controlled trials, with each subject receiving one dose of either drug or placebo. All patients had a preliminary screening visit at which the diagnosis was confirmed. Patients must have had low actual or functional amounts of plasma C1 inhibitor and low C4 with a normal C1q. There were also details that differed between the studies. For example, the type of attack acceptable for the treatment protocol varied from study to study: some allowed peripheral edema attacks, but some did not, and some allowed facial attacks, but some did not. For some studies, the FDA allowed C1 inhibitor to be used as the rescue medication. In all studies the C1 inhibitor was administered intravenously. The only prophylaxis trial had a crossover design, as discussed below. The Behring study examined responses to abdominal and facial attacks, and the Lev study examined responses to abdominal, facial, and genital attacks. In the Lev study, the product, now called Cinryze, was used at 1000 units per patient with the possibility of the dose being repeated if the patient did not respond[22]. In the Behring study, the product, now known as Berinert, was used at either 10 or 20 units/kg[23]. In the Pharming study the product was used at 50 or 100 units/kg[21]; a higher dose was used because the recombinant protein has a much shorter half-life in the circulation than the plasma-derived protein. This is presumably because the rabbit glycosylates proteins differently from humans, and the C1 inhibitor is a heavily glycosylated protein.

**Figure 1:**
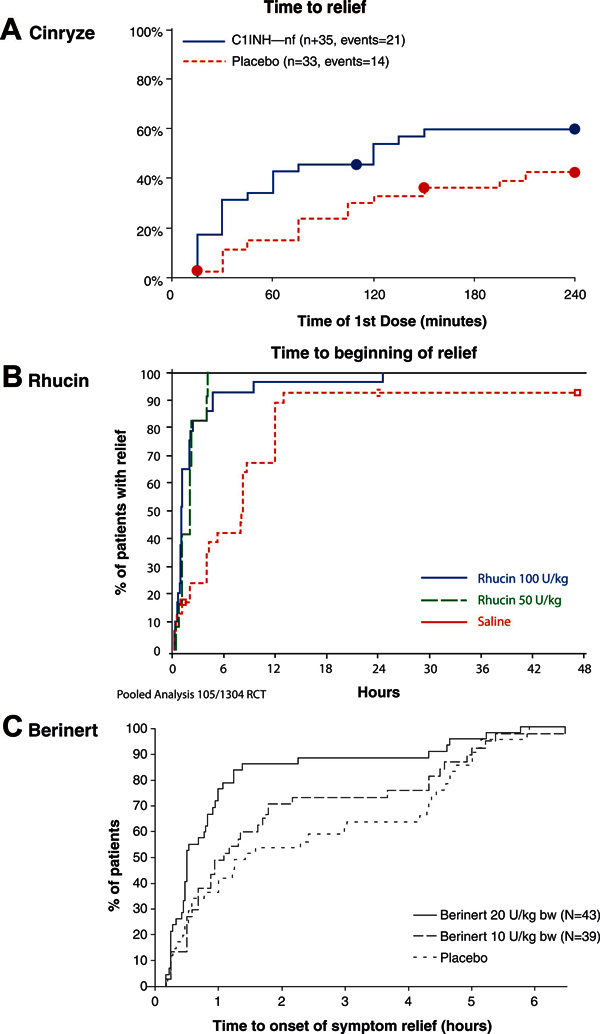
**Two plasma-derived C1 inhibitor preparations, Cinryze (A) and Berinert (C), and a recombinant protein, Rhucin (B), provide faster symptom relief for acute HAE attacks than placebo**. Reprinted with permission from (A) *New England Journal of Medicine*,[22], (B) *Pharming Group NV, (C) Journal of Allergy and Clinical Immunology*[23].

All 3 preparations of C1 inhibitor seem to be effective in terminating attacks of angioedema (Figure 1). Response is dose-dependent, and it is likely that small differences are determined by differences in the dose administered and the severity and type of attack. Dose-finding studies were conducted by Pharming (personal communication) and CSL Behring[23]. Patients with moderate disease started to recover in a mean of about 9 hours with placebo and recovered in about 5 hours when given C1 inhibitor. CSL Behring found that 20 units/kg Berinert, not 10 units/kg, was significantly effective in terminating attacks. Although fewer patients in this group were studied, with severe disease the difference was even more striking with severe attacks, which showed relief in 12 hours with placebo and in 2 hours with C1 inhibitor. Patients responded to both the 50 and 100 unit/kg dose of Rhucin. In general, abdominal and throat attacks responded much more rapidly than peripheral attacks. This may be a reflection of the fact that resorption of peripheral edema is slow. The amount of edema that it takes to compromise the airway is small and the amount of edema that it takes to stretch the gut mucosa is relatively small or relatively easily absorbed in this area of high vascularity. In earlier studies, clear responses were seen at 30 minutes, but in no case did the agent lead to complete recovery in this time frame[19]. All the patients receiving placebo also recovered; HAE is a self-limited disease and in all cases attacks resolve spontaneously[1,2]. Part of the speed of response is determined by the stage of attack when the patient is treated. If the patient is treated early in an attack, resolution may be quick. If he or she is at the peak of the attack, resolution may occur more slowly. If the patient has started to recover and is no longer generating bradykinin, the recovery may be rapid. There is no biochemical test that allows the physician to grade the severity of an angioedema attack or to determine whether an attack is becoming more severe or is in the recovery phase, and so many of these decisions are based on experience and judgment.

It appears that both of the plasma-derived products, Cinryze and Berinert, are effective in acute therapy. The Behring product was approved by the FDA for treatment of acute attacks of HAE in October 2009. The recombinant product is reported to be effective but has not yet been approved for therapy.

## Prophylaxis of Attacks

Perhaps 20% of patients have attacks that are sufficiently frequent or sufficiently severe that prophylaxis is needed. As noted above, the drugs that have been traditionally used for prophylaxis are not suitable in all situations and are associated with side effects that in some patients may be intolerable. In October 2008, the C1 inhibitor preparation Cinryze (Viropharma) was approved by the FDA for prophylaxis against HAE angioedema attacks. Approval followed a double-blind study that had a crossover design[22]. Patients received 1000 units of Cinryze or an equal volume of placebo as an intravenous infusion 2 times per week. If the patient's symptoms were not controlled on 2 infusions per week, a third infusion of Cinryze as open-label treatment was permitted. After 12 weeks of treatment with drug or placebo, there was a crossover so that patients receiving drug were switched to placebo and patients on placebo were switched to drug. The number, location, duration, and severity of attacks were recorded, as was the number of times that rescue medication was administered. The results were clear cut: on all of these parameters patients did better on drug, and the results were statistically highly significant. Most patients had fewer attacks, although attacks were not eliminated altogether, but in some patients the attack frequency did decrease to zero. One patient had a markedly increased frequency of attacks on Cinryze compared with placebo. These results point out several things. In none of these studies did the amount of drug given lead to complete normalization of C1 inhibitor levels, and it seems reasonable to expect that in some patients attacks may continue. It had previously been noted before that there is no relationship between C1 inhibitor levels and the incidence or severity of attacks[1]. Patients with relatively high levels of C1 inhibitor may have severe and frequent attacks, and patients with relatively low levels may have mild disease. Because the level of bradykinin is determined not only by the rate of generation of the peptide but also by its rate of degradation, it is reasonable to hypothesize, but has not been shown experimentally, that differences in the rate of bradykinin degradation may explain this finding. Psychologic factors also have a marked influence on the frequency and severity of attacks[22]. The individual with increased attack frequency had a series of major personal problems during the 12-week period when she was receiving Cinryze. During the period when she was receiving placebo her life situation was much improved and her attack frequency was much lower.

## Advantages and Disadvantages of the Various Agents

There are theoretical problems with all products. The plasma products carry some risk of infection. Hepatitis was transmitted by plasma-purified C1 inhibitor in the very early days of its use, but with the introduction of more highly refined purification and pasteurization techniques there have been no cases of infectious disease transmission by these agents for decades, and they are used extensively in Europe. Plasma-derived inhibitors have the advantage that because all patients are heterozygous for deficient C1 inhibitor function, they have one normal gene producing normal secreted protein, and allergy to the administered product is unlikely unless it is modified during the purification. Furthermore, because this is the normal physiologic protein, it is expected to work in the normal way to restore patients to physiologic balance.

Recombinant C1 inhibitor carries less risk of infection, although rabbits do have some retroviruses. However, glycosylation of the recombinant protein differs from that of the normal C1 inhibitor, resulting in a shorter half-life. As a result, it is unlikely to be of use in prophylaxis. Because of the difference in sugars, there is some risk of allergy; although some allergy has been noted to rabbit proteins, the author is not aware of any allergy to the purified human C1 inhibitor. Because it is not a serum product, in theory the supply is limitless. All of the reported testing has used intravenous administration and only intravenous products are FDA-approved. This limits self-administration and is therefore inconvenient for the patient.

## Cost of the New Therapies for Hereditary Angioedema

HAE has been designated an "Orphan Disease", meaning that there are so few patients that a drug manufacturer is unlikely to see the development of therapy as profitable. For that reason the U.S. Congress some years ago gave manufacturers an incentive to develop drugs for this group of diseases by providing for 7 years of exclusivity; that is, the development of new effective therapy for an Orphan Disease guarantees the developers that no other manufacturer will be able to market the same product for 7 years. This has led directly to an effort to have new drugs approved for the treatment of HAE for the American market. Interestingly, multiple approaches to this problem have led to the almost simultaneous development of effective agents. Development and testing of drugs is expensive and the developers want to assure their investors of a profit. As is often the case with new drugs developed through the Orphan Disease program, the drugs are expensive. Thus far, we know the cost of the plasma-derived C1 inhibitor products and of Ecallantide. All are marketed at well over $1000 dollars a dose and are far more expensive than danazol, but all seem to have a better safety profile. With so many new effective agents on the market, it is not clear how pricing will proceed in the future.

## Conclusions

The development of C1 inhibitor prophylaxis and therapy for acute treatment represents an enormous milestone in the history of HAE treatment. For the first time, Americans have drugs available that clearly will terminate attacks. Furthermore, there is now effective prophylactic therapy as well. It is likely that some patients will continue to take the androgens for cost reasons and because they are relatively low in toxicity. Clearly, the toxicity profile of the new agents is preferable. Nevertheless, pregnant women and children have therapeutic alternatives that were not available in this country in the past, and their lives will be greatly improved.
